# Identification and Monitoring of *Lactobacillus delbrueckii* Subspecies Using Pangenomic-Based Novel Genetic Markers

**DOI:** 10.4014/jmb.2009.09034

**Published:** 2020-10-22

**Authors:** Eiseul Kim, Eun-Ji Cho, Seung-Min Yang, Hae-Yeong Kim

**Affiliations:** Institute of Life Sciences and Resources and Department of Food Science and Biotechnology, Kyung Hee University, Yongin 17104, Republic of Korea

**Keywords:** *Lactobacillus delbrueckii* subspecies, pangenome, genetic marker, identification, real-time PCR, probiotic product

## Abstract

Genetic markers currently used for the discrimination of *Lactobacillus delbrueckii* subspecies have low efficiency for identification at subspecies level. Therefore, our objective in this study was to select novel genetic markers for accurate identification and discrimination of six *L. delbrueckii* subspecies based on pangenome analysis. We evaluated *L. delbrueckii* genomes to avoid making incorrect conclusions in the process of selecting genetic markers due to mislabeled genomes. Genome analysis showed that two genomes of *L. delbrueckii* subspecies deposited at NCBI were misidentified. Based on these results, subspecies-specific genetic markers were selected by comparing the core and pangenomes. Genetic markers were confirmed to be specific for 59,196,562 genome sequences via in silico analysis. They were found in all strains of the same subspecies, but not in other subspecies or bacterial strains. These genetic markers also could be used to accurately identify genomes at the subspecies level for genomes known at the species level. A real-time PCR method for detecting three main subspecies (*L. delbrueckii* subsp. *delbrueckii*, *lactis*, and *bulgaricus*) was developed to cost-effectively identify them using genetic markers. Results showed 100% specificity for each subspecies. These genetic markers could differentiate each subspecies from 44 other lactic acid bacteria. This real-time PCR method was then applied to monitor 26 probiotics and dairy products. It was also used to identify 64 unknown strains isolated from raw milk samples and dairy products. Results confirmed that unknown isolates and subspecies contained in the product could be accurately identified using this real-time PCR method.

## Introduction

*Lactobacillus delbrueckii* comprises six subspecies, namely *delbrueckii*, *lactis*, *bulgaricus*, *indicus*, *jakobsenii*, and *sunkii*, all of which have historically been differentiated based on their ability to metabolize different carbohydrates [1]. Among these subspecies, *L. delbrueckii* subsp. *lactis* and *bulgaricus* are usually associated with the manufacture of dairy products such as cheeses and yogurt [2]. *L. delbrueckii* subsp. *bulgaricus* is one of the starter culture components for the production of yogurt [1, 3]. This subspecies displays probiotic properties [4]. On the other hand, *L. delbrueckii* subsp. *lactis* is traditionally used in cheese production and can be distinguished from *L. delbrueckii* subsp. *bulgaricus* by its extensive carbohydrate metabolizing capabilities [1, 5]. *L. delbrueckii* subsp. *delbrueckii* cannot ferment lactose. It is typically associated with fermented vegetables [2]. *L. delbrueckii* subsp. *indicus*, *jakobsenii*, and *sunkii* are relatively minor subspecies isolated from Indian dairy products, fermented alcoholic beverages, and non-salted pickle as a traditional Japanese food, respectively [6-9].

Accurate identification of *L. delbrueckii* subspecies in food samples is an important issue to confirm probiotic properties and perform product quality assessment [4]. Genetic markers and molecular-based methods have been used to efficiently identify and detect lactic acid bacteria commonly used in commercial probiotic and dairy products. Molecular-based methods for the identification and typing of lactic acid bacteria have been reported, including amplified fragment length polymorphism (AFLP), DNA–DNA hybridization (DDH), multi-locus sequence analysis (MLST), and restriction fragment length polymorphism (RFLP) [6,10-12]. However, these techniques are labor-intensive, expensive, and time-consuming with low reproducibility whereas PCR-based methods are rapid, sensitive, and reliable for identifying lactic acid bacteria [4]. Of these methods, genetic markers such as the 16S rRNA gene and 16S–23S rRNA intergenic spacer region have been used to distinguish *L. delbrueckii* used in PCR-based methods [13]. Although genetic markers described above are useful for identifying *L. delbrueckii* at the species level, they cannot be applied to distinguish *L. delbrueckii* at the subspecies level [4].

Recently, the development of whole-genome sequencing (WGS) and the increase in genome sequences have made it possible to rapidly and freely process large-scale sequence data on microorganisms in public repositories [14]. Pangenome analysis based on WGS has a wide range of applications, including prediction of lifestyles of microorganisms, pathogenicities, resistome, and taxonomy [15]. Pangenome analysis also allows reclassification of bacterial species and/or subspecies, improving and clarifying criteria previously presented [16]. In the present study, we selected six *L. delbrueckii* subspecies-specific genetic markers by pangenome analysis to develop a real-time PCR method for rapid identification of bacterial strains. The real-time PCR method we developed was then applied to bacterial strains isolated from raw milk, probiotic products, and dairy products to identify and differentiate three *L. delbrueckii* subspecies.

## Materials and Methods

### Pangenome Analysis and Selection of Genetic Markers

**Fig. 1:**
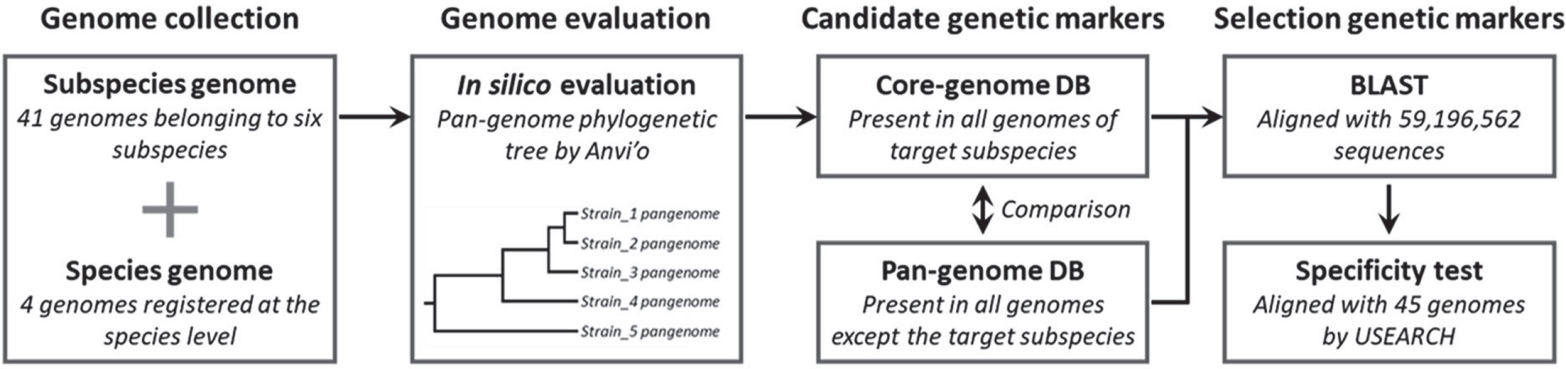
The in silico approach for genetic marker selection of six *L. delbrueckii* subspecies.

The in silico scheme for selecting the genetic markers of six *L. delbrueckii* subspecies is illustrated in Fig. 1. A total of 41 genomes belonging to the subspecies *L. delbrueckii* subsp. *delbrueckii*, *lactis*, *bulgaricus*, *indicus*, *jakobsenii*, and *sunkii* were obtained from the National Center for Biotechnology Information (NCBI) (Table 1). Phylogenetic analysis based on the pangenome was performed using microbial pangenomics in Anvi’o v6 software [17]. According to the developer’s recommendation, a genome database for pangenome analysis was constructed using Anvi’o genome storage. The pangenome was then analyzed using the NCBI BLASTp and MCL algorithm. Subsequently, a phylogenetic tree was constructed based on pangenome cluster frequencies.

The pangenome of *L. delbrueckii* subspecies was calculated using Bacterial Pan Genome Analysis (BPGA) pipeline ver. 1.3 (identity cut off = 50%) [18]. The pangenome was formatted into two local databases: a pangenome database and a core-genome database for each subspecies. Candidate genetic markers were selected by comparing the pangenome database composed of protein-coding genes, present in all genomes except for the target subspecies, and the core-genome database composed of protein-coding genes present in all genomes of target subspecies. Candidate genetic markers were then aligned with 59,196,562 sequences using BLASTN. Genetic markers only present in target subspecies but not present in other bacterial genomes were selected.

### In Silico Specificity Confirmation and Development of Subspecies-Specific Primer

A total of four genome sequences registered at the species level were obtained from the NCBI. Genetic marker specificity was aligned with 45 genome sequences, including genomes known to the species level using USEARCH ver. 9.0 [19]. Alignment results are presented as a heatmap using Seaborn python library in Matplotlib. Subspecies-specific primer pairs for *L. delbrueckii* subsp. *lactis*, *bulgaricus*, and *delbrueckii* were designed from their genetic markers using Primer Designer (Scientific and Education Software, USA).

### DNA Extractions from *L. delbrueckii* Subspecies and Lactic Acid Bacteria

For specificity testing of primer pairs developed in this study, the reference strains of lactic acid bacteria mainly isolated from probiotic and dairy products were used. A total of 54 strains of lactic acid bacteria including *L. delbrueckii* subspecies were obtained from the Korean Agricultural Culture Collection (KACC, Korea), the Korean Collection for Type Cultures (KCTC, Korea), the Korean Culture Center of Microorganisms (KCCM, Korea), the NITE Biological Resource Center (NBRC, Japan), and the Laboratory Isolates (LI, Korea) (Table 2). All reference strains were grown in MRS broth (Difco, Becton & Dickinson, USA) for extraction of genomic DNA. *L. delbrueckii* and other bacterial strains were cultured for 48 h at 42°C and 37°C under anaerobic condition, respectively. Bacterial cells were centrifuged at 13,600 ×*g* for 5 min and the supernatant was removed. Genomic DNA of reference strains was extracted using a DNeasy Blood & Tissue Kit (Qiagen, Germany) following the protocol described previously [13, 20]. DNA concentration and purity were confirmed using a MaestroNano spectrophotometer (Maestrogen, USA).

### Specificity and Accuracy of Specific Primer Pairs

Real-time PCR assay was conducted to determine the specificity and accuracy of primer pairs using a 7500 Real-time PCR system. Each reaction contained 20 ng of genomic DNA, 10 μl of 2X Thunderbird SYBR qPCR Mix (Toyobo, Japan), 500 nM of primer pairs, and distilled water up to 20 μl total volume. Real-time PCR conditions consisted of initiation at 95°C for 2 min followed by 30 amplification cycles of 95°C for 5 s and 60°C for 30 s. Melting curves were obtained at 95°C for 15 s, 60°C for 1 min, 95°C for 30 s, and 60°C for 15 s. Specificity of primer pairs was tested against a total of 10 strains of *L. delbrueckii* subspecies and 44 other lactic acid bacteria. For the accuracy test, genomic DNA from each reference strain was serially diluted and used for real-time PCR.

### Application of Real-Time PCR Method

To test the developed real-time PCR method, 64 isolates, 15 probiotic products, and 11 dairy products were used. *L. delbrueckii* subspecies were isolated from three raw milk samples and three dairy products. Serially diluted samples were spread onto MRS agar plates (Difco, Becton & Dickinson, USA) and incubated at 42°C for 48 h under anaerobic conditions. Probiotic and dairy products were obtained from markets around the world. Genomic DNAs were extracted from isolates and products under the same conditions as described in section 2.3. DNA Extraction of *L. delbrueckii* Subspecies and Lactic Acid Bacteria. For the application test, genomic DNAs of isolates or products were added to wells of 96-well plates containing 2X qPCR mix (Toyobo) and subspecies-specific primer pairs. The real-time PCR condition was the same as that described in section 2.4. Specificity and Accuracy for Specific Primer Pairs.

## Results and Discussion

### Pangenome Analysis

**Fig. 2:**
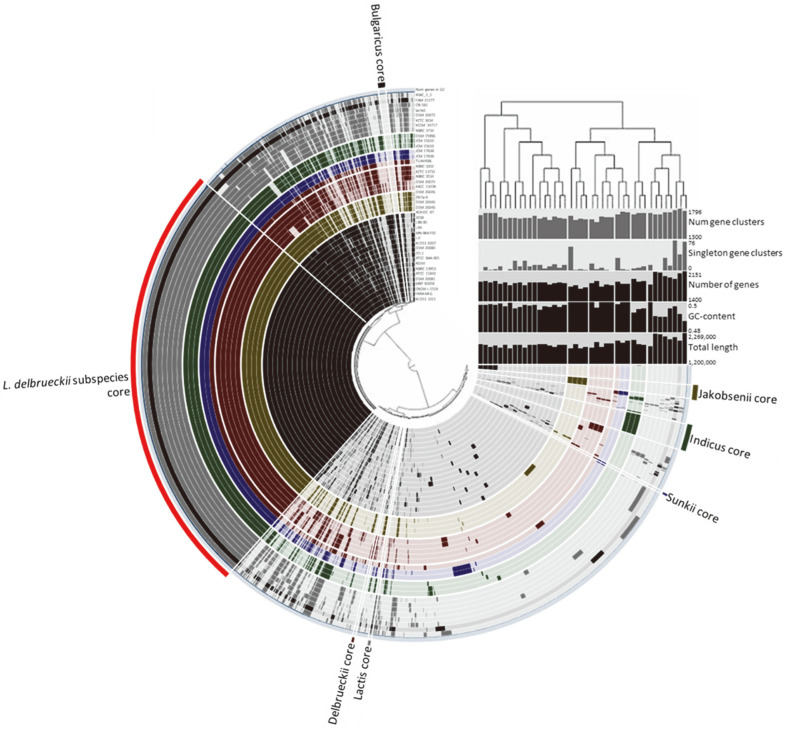
Pangenome distribution of the 41 *L. delbrueckii* subspecies genomes. The color bar of black, yellow, red, blue, green, and green represents *L. delbrueckii* subsp. *bulgaricus*, *jakobsenii*, *delbrueckii*, *sunkii*, *indicus*, and *lactis* genomes, respectively. The dark color and tinted bright of the bar indicate core-genome presence and absence, respectively. The phylogenetic tree on the right is based on gene cluster frequencies.

Many studies have previously reported a mislabeling issue regarding subspecies or closely related species in the NCBI genome database [20, 21]. In these studies, the majority of the mislabeled genomes were closely related species [20, 22, 23]. Such genomes should therefore be evaluated to avoid reaching incorrect conclusions in a comparative genomic analysis. In the present study, for the first time, we evaluated the genomes of *L. delbrueckii* subspecies by phylogenetic analysis based on the pangenome before specific genetic markers were selected. Phylogenetic analysis results based on pangenome frequencies were displayed along with the distribution of subspecies’ specific regions. Each bar represents *L. delbrueckii* subspecies genomes and each layer presents pangenome distribution (Fig. 2). Most genomes clustered according to the subspecies. However, some genomes of *L. delbrueckii* subsp. *bulgaricus* and *delbrueckii* clustered with different subspecies. Genomes of *L. delbrueckii* subsp. *bulgaricus* FAM 21277 and *delbrueckii* TUA4408L clustered with *L. delbrueckii* subsp. *lactis* and *sunkii*, respectively. Based on these results, *L. delbrueckii* subsp. *bulgaricus* FAM 21277 and *delbrueckii* TUA4408L were determined as *L. delbrueckii* subsp. *lactis* and *sunkii*, respectively. These genomes should be indicated correctly in the genome database to avoid further misidentification. We also suggest implementing measures to prevent and correct taxonomic errors in the NCBI database to avoid confusion in future research.

Closely related strains in phylogenetic analysis can be distinguished using efficient and customized mining methods for genome sequences [20, 24, 25]. Conventional methods can be used to successfully distinguish pathogenic bacteria that are difficult to differentiate, although these methods only focus on pathogenic bacteria. Studies on lactic acid bacteria are still lacking. Here, we employed a pangenome approach to identify novel genetic markers for specific identification and detection of *L. delbrueckii* subspecies.

As a result of pangenome analysis, a total of 67,178 genes from 41 *L. delbrueckii* subspecies yielded a pangenome size of 3,456 genes. The core-genome, accessory-genome, and unique-genome had 749, 2,071, and 636 genes, respectively. Six subspecies-specific genetic markers were then obtained by pangenome analysis. Genetic markers were found to be protein-coding genes present in the same subspecies but absent in other subspecies or bacterial strains. By comparing genomes of the same subspecies, 995 to 1,628 protein-coding genes were found in common in the genomes of each subspecies and considered as the core-genome for each subspecies. After comparing each core-genome with pangenome for protein-coding genes present in all genomes except for target genomes, 5 to 50 protein-coding genes were selected as candidate genetic markers for each subspecies. These candidate genetic markers were aligned with 59,196,562 genome sequences. Genes not present in other bacterial strains except target subspecies were finally selected as genetic markers. Selected genetic markers of *L. delbrueckii* subsp. *bulgaricus*, *lactis*, *delbrueckii*, *indicus*, *jakobsenii*, and *sunkii* were identified as YcaO-like family protein (Accession No. ABJ57813.1), Ser/Thr protein kinase (Accession No. EGD27260.1), choline kinase (Accession No. KNZ37552.1), DNA methyltransferase (Accession No. KNE31255.1), RpoD family RNA polymerase sigma factor (Accession No. EOD03403.1), and hypothetical protein (Accession No. APG74821.1), respectively.

### Genetic Marker Specificity Test

**Fig. 3:**
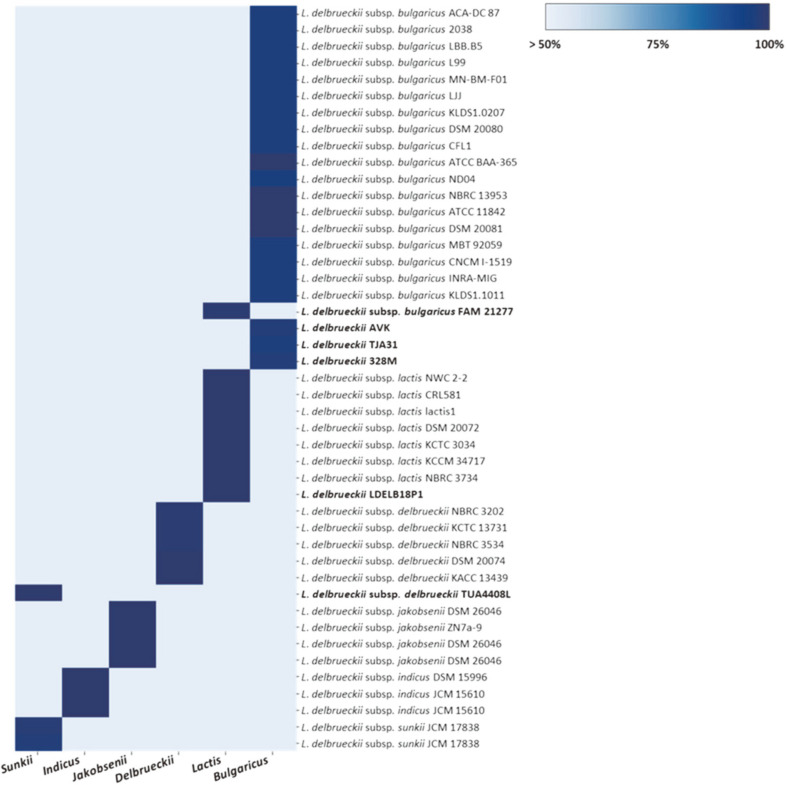
Heatmap shows the presence/absence of genetic markers in 45 genomes. The heatmap shows that the similarity of genetic markers present in each genome is visualized with a color bar of blue (high identity) to sky blue (low identity). The bottom of the heatmap presents the six subspecies-specific genetic markers; the right of the heatmap presents the 45 genomes of *L. delbrueckii* species or subspecies.

The specificity of genetic markers was tested using 45 genomes including genomes registered at species level by in silico analysis. The heatmap for identities of genetic markers in genomes is shown with color codes, ranging from blue (region with high identity) to sky blue (region with low identity) (Fig. 3). Each genetic marker shared more than 95% sequence identity with genomes of most corresponding subspecies. In contrast, a genetic marker for *L. delbrueckii* subsp. *bulgaricus* was present in 19 *bulgaricus* genomes (95–100% identity), but one genome had the genetic marker for *L. delbrueckii* subsp. *lactis* instead of *bulgaricus* (99% identity). A genetic marker for *L. delbrueckii* subsp. *delbrueckii* was present in six *delbrueckii* genomes (99–100% identity), but one genome had the genetic marker for *L. delbrueckii* subsp. *sunkii* instead of *delbrueckii* (100% identity). These results were the same as those of pangenome analysis. Genetic markers were aligned with their genomes to determine the subspecies of genomes registered at the species level. *L. delbrueckii* AVK, TJA31, and 328M contained the genetic marker for *L. delbrueckii* subsp. *bulgaricus* (96–97% sequence identity). *L. delbrueckii* LDELB18P1 contained the genetic marker for *L. delbrueckii* subsp. *lactis* (100% sequence identity).

In previous studies, genes such as 16S rRNA, 16S–23S rRNA intergenic spacer region, and the elongation factor Tu (tuf) gene have been used to distinguish microorganisms at the species or subspecies level [4,9,26-29]. However, some studies have reported that these genes share high sequence similarities without showing sufficient variabilities to allow for the differentiation between *L. delbrueckii* subspecies [4, 13]. In contrast, we selected genetic markers specific to the genomes of each subspecies using pangenome analysis. The markers selected in this study were specific to *L. delbrueckii* subspecies and other bacterial strains. They were able to accurately identify subspecies level for unknown genomes.

### Specificity and Accuracy for Specific Primer Pairs

The method to identify *L. delbrueckii* subspecies with genetic markers selected in this study requires WGS and bioinformatics analysis to confirm the presence of their markers. This method can accurately identify *L. delbrueckii* subspecies. However, the cost associated with WGS and its informational capacities must be considered. In addition, specialized researchers are needed to handle bioinformatics analysis [14, 30]. Therefore, we developed a real-time PCR method to cost-effectively identify many *L. delbrueckii* isolates using relatively simple procedures. This real-time PCR method is designed to identify three main subspecies, *L. delbrueckii* subsp. *delbrueckii*, *lactis*, and *bulgaricus* [2] that are mainly isolated from food or used for fermenting dairy products.

**Fig. 4:**
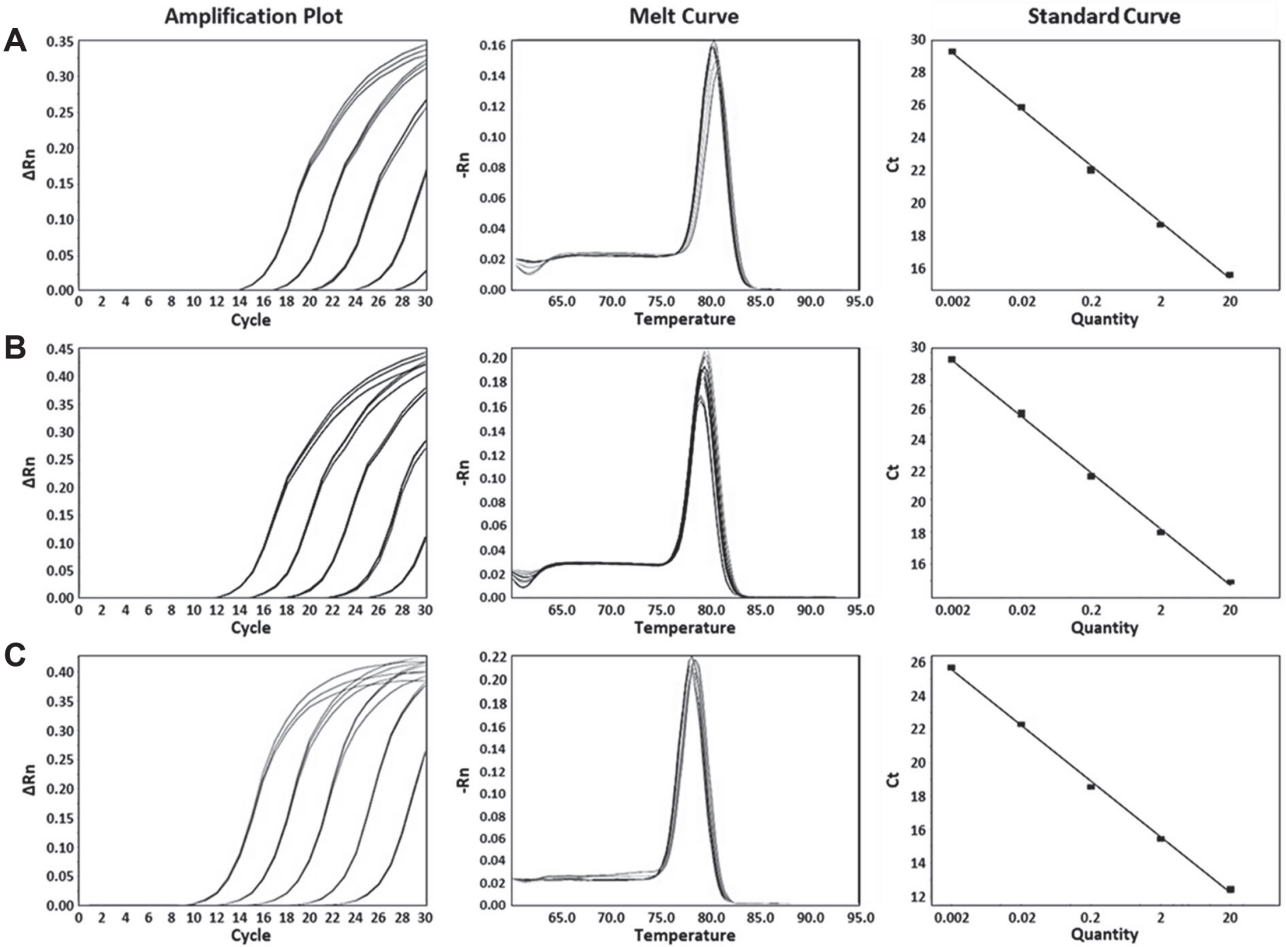
The specificity of subspecies-specific primer pairs against 54 lactic acid bacteria. (**A**) Specificity of *L. delbrueckii* subsp. *bulgaricus* primer pair, amplification curve: *L. delbrueckii* subsp. *bulgaricus* KACC 12420, LI 00010, LI 00011, LI 00012, LI 00013, and LI 00014; (**B**) Specificity of *L. delbrueckii* subsp. *lactis* primer pair, amplification curve: *L. delbrueckii* subsp. *lactis* KACC 12417 and LI 00015; (**C**) Specificity of *L. delbrueckii* subsp. *delbrueckii* primer pair, amplification curve: *L. delbrueckii* subsp. *delbrueckii* KACC 13439 and KCTC 13730.

**Fig. 5:**
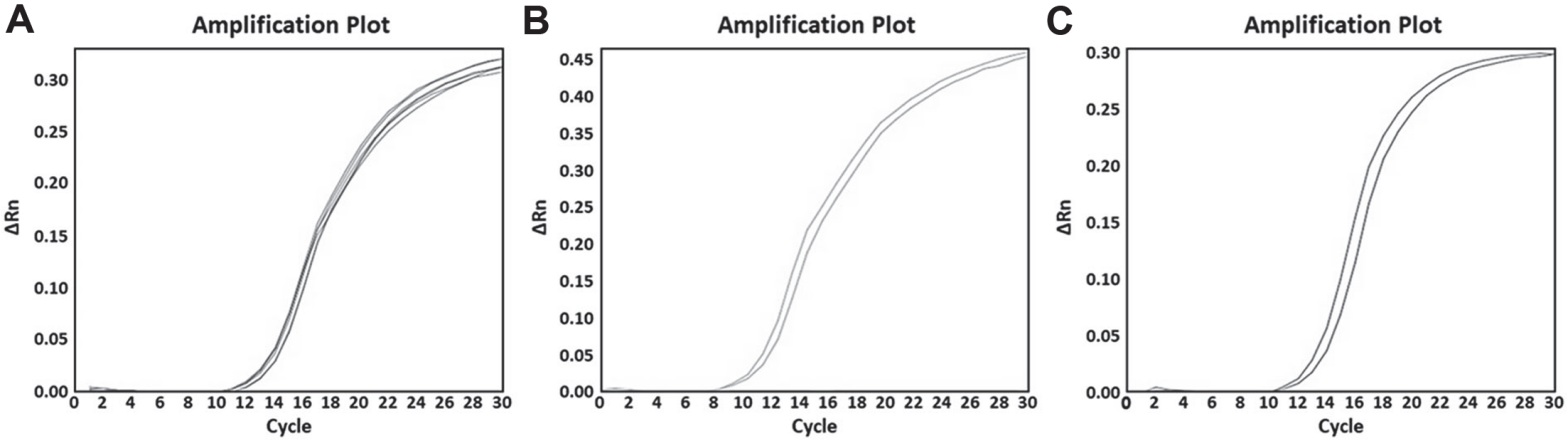
Real-time PCR amplification plot, melt curve, and standard curve. (**A**) *L. delbrueckii* subsp. *bulgaricus* amplification plot (left), melt curve (middle), and standard curve (right); (**B**) *L. delbrueckii* subsp. *lactis* amplification plot (left), melt curve (middle), and standard curve (right); (**C**) *L. delbrueckii* subsp. *delbrueckii* amplification plot (left), melt curve (middle), and standard curve (right).

Subspecies-specific primer pairs were designed from selected genetic markers. Information for primer pairs is shown in Table 3. The specificity test for these designed subspecies-specific primer pairs was performed using 54 reference strains of lactic acid bacteria. The genomic DNA of each subspecies generated a positive signal for corresponding primer pairs, whereas genomic DNAs from other *L. delbrueckii* subspecies and lactic acid bacteria did not generate any signal (Fig. 4). The Ct value ranged from 12.72 to 16.94 for each subspecies-specific primer pair. Genomic DNAs of three subspecies were used to confirm the accuracy of primer pairs. Standard curves were generated using serial diluted genomic DNA at an amount ranging from 0.002 ng to 20 ng. Slopes for standard curves of *L. delbrueckii* subsp. *bulgaricus*, *lactis*, and *delbrueckii* were −3.44, −3.46, and −3.34, respectively. All correlation coefficient values (*R*^2^) were greater than 0.998 and all amplification efficiencies were more than 94%(Fig. 5). All of these values met real-time PCR conditions indicating a high efficiency [31]. Thus, the method we developed in the present study shows high accuracies. Our real-time PCR method targeting specific genetic markers enables rapid and accurate identification of three *L. delbrueckii* subspecies.

### Application of Real-Time PCR Method

Fifty-one isolates, 15 probiotic products, and 11 dairy products were used to perform the application test of the developed real-time PCR method. Results of its application to probiotic and dairy products were compared with their label claims. A total of 26 products were detected with the same subspecies as their label claims (Table 4). However, for 16 products, subspecies was incorrectly claimed on the label. According to the nomenclature of subspecies, these should be labeled as “*L. delbrueckii* subsp. *bulgaricus*,” not “L. *bulgaricus*” [12]. Dairy products labeled only with lactic acid bacteria were confirmed to contain *L. delbrueckii* subsp. *bulgaricus* by real-time PCR. As a result of the application of our method to different isolates, a total of 64 strains isolated from raw milk and dairy products were identified as *L. delbrueckii* subsp. *lactis* (*n* = 17) and *bulgaricus* (*n* = 47) (Table 4). These results confirmed that the real-time PCR developed in this study could accurately identify strains present in probiotic and dairy products and bacterial isolates to the subspecies level.

## Conclusion

In conclusion, pangenome analysis was performed to select genetic markers for six *L. delbrueckii* subspecies. These genetic markers were present in all genomes of the same subspecies but absent in genomes of other subspecies and bacterial strains. To rapidly and cost-effectively identify *L. delbrueckii* subspecies, subspecies-specific primer pairs for three subspecies mainly isolated from food samples were designed. The real-time PCR method using these genes could accurately identify *L. delbrueckii* subspecies and other lactic acid bacteria with high specificity. The developed real-time PCR method was able to successfully monitor probiotic and dairy products and identify various isolates. Thus, our method can be used to accurately identify *L. delbrueckii* subspecies and determine the nomenclature of these subspecies. Furthermore, it can contribute to safety in the food industry by ensuring products are labeled to show the correct strain.

## Figures and Tables

**Table 1:** Summary in genome features of 41 *L. delbrueckii* subspecies.

Organism name	Strain	Size (Mb)	GC%	CDS	Assembly	Accession no.
*L. delbrueckii* subsp. *bulgaricus*	ATCC BAA-365	1.85695	49.7	1579	Complete	CP000412.1
*L. delbrueckii* subsp. *bulgaricus*	ATCC 11842	1.865	49.7	1561	Complete	CR954253.1
*L. delbrueckii* subsp. *bulgaricus*	2038	1.87292	49.7	1562	Complete	CP000156.1
*L. delbrueckii* subsp. *bulgaricus*	CNCM I-1519	1.79654	49.9	1630	Contig	AGHW01
*L. delbrueckii* subsp. *bulgaricus*	INRA-MIG	1.85324	49.8	1692	Scaffold	CCET01
*L. delbrueckii* subsp. *bulgaricus*	DSM 20081	1.75853	49.9	1533	Scaffold	JQAV01
*L. delbrueckii* subsp. *bulgaricus*	MN-BM-F01	1.87507	49.7	1585	Complete	CP013610.1
*L. delbrueckii* subsp. *bulgaricus*	CFL1	1.75792	49.8	1539	Contig	CZPS01
*L. delbrueckii* subsp. *bulgaricus*	LBB.B5	1.77788	49.8	1558	Contig	LUGK01
*L. delbrueckii* subsp. *bulgaricus*	DSM 20080	1.86818	49.8	1564	Complete	CP019120.1
*L. delbrueckii* subsp. *bulgaricus*	ND04	1.86175	49.6	1538	Complete	CP016393.1
*L. delbrueckii* subsp. *bulgaricus*	MBT 92059	1.83117	49.8	1648	Scaffold	QOVB01
*L. delbrueckii* subsp. *bulgaricus*	L99	1.84811	49.7	1575	Complete	CP017235.1
*L. delbrueckii* subsp. *bulgaricus*	KLDS1.0207	1.86918	49.8	1620	Complete	CP032451.1
*L. delbrueckii* subsp. *bulgaricus*	FAM 21277	2.01984	49.2	1830	Contig	VBSR01
*L. delbrueckii* subsp. *bulgaricus*	NBRC 13953	1.72582	50.0	1519	Contig	BJMY01
*L. delbrueckii* subsp. *bulgaricus*	KLDS1.1011	1.88749	49.8	1634	Complete	CP041280.1
*L. delbrueckii* subsp. *bulgaricus*	LJJ	1.89109	49.5	1604	Complete	CP049052.1
*L. delbrueckii* subsp. *bulgaricus*	ACA-DC 87	1.856	49.8	1579	Complete	LT899687.1
*L. delbrueckii* subsp. *delbrueckii*	KACC 13439	1.76619	50.0	1485	Contig	LHPL01
*L. delbrueckii* subsp. *delbrueckii*	KCTC 13731	1.91051	50.0	1600	Complete	CP018216.1
*L. delbrueckii* subsp. *delbrueckii*	DSM 20074	1.95372	49.6	1577	Complete	CP018615.1
*L. delbrueckii* subsp. *delbrueckii*	TUA4408L	2.01244	49.9	1718	Complete	CP021136.1
*L. delbrueckii* subsp. *delbrueckii*	NBRC 3534	1.78742	50.3	1588	Contig	BJLM01
*L. delbrueckii* subsp. *delbrueckii*	NBRC 3202	1.91031	50.1	1653	Complete	AP019750.1
*L. delbrueckii* subsp. *indicus*	JCM 15610	1.87741	49.5	1627	Contig	LGAS01
*L. delbrueckii* subsp. *indicus*	DSM 15996	1.86357	49.6	1621	Scaffold	AZFL01
*L. delbrueckii* subsp. *indicus*	JCM 15610	2.02186	49.4	1694	Complete	CP018614.1
*L. delbrueckii* subsp. *jakobsenii*	ZN7a-9	1.73081	50.2	1552	Contig	ALPY01
*L. delbrueckii* subsp. *jakobsenii*	DSM 26046	1.74924	50.3	1568	Scaffold	JQCG01
*L. delbrueckii* subsp. *jakobsenii*	DSM 26046	1.8918	50.1	1614	Complete	CP018218.1
*L. delbrueckii* subsp. *jakobsenii*	DSM 26046	1.78119	50.1	1585	Scaffold	PUFG01
*L. delbrueckii* subsp. *lactis*	CRL581	2.13682	49.6	1639	Scaffold	ATBQ01
*L. delbrueckii* subsp. *lactis*	KCCM 34717	2.26338	49.1	1905	Complete	CP018215.1
*L. delbrueckii* subsp. *lactis*	DSM 20072	2.16598	49.0	1793	Complete	CP022988.1
*L. delbrueckii* subsp. *lactis*	KCTC 3034	2.23761	49.0	1889	Complete	CP023139.1
*L. delbrueckii* subsp. *lactis*	NBRC 3734	1.81291	50.2	1653	Contig	BJLO01
*L. delbrueckii* subsp. *lactis*	lactis1	2.05032	49.6	1694	Complete	LS991409.1
*L. delbrueckii* subsp. *lactis*	NWC_2_2	2.269179	48.7	1934	Complete	CP031023.1
*L. delbrueckii* subsp. *sunkii*	JCM 17838	1.94526	50.1	1713	Contig	LGHR01
*L. delbrueckii* subsp. *sunkii*	JCM 17838	2.00434	50.1	1726	Complete	CP018217.1

**Table 2:** List of reference strains used in this study.

Species	Strain no.
*Lactobacillus delbrueckii* subsp. *bulgaricus*	KACC^a^ 12420
*Lactobacillus delbrueckii* subsp. *bulgaricus*	LI^b^ 00010
*Lactobacillus delbrueckii* subsp. *bulgaricus*	LI 00011
*Lactobacillus delbrueckii* subsp. *bulgaricus*	LI 00012
*Lactobacillus delbrueckii* subsp. *bulgaricus*	LI 00013
*Lactobacillus delbrueckii* subsp. *bulgaricus*	LI 00014
*Lactobacillus delbrueckii* subsp. *lactis*	KACC 12417
*Lactobacillus delbrueckii* subsp. *lactis*	LI 00015
*Lactobacillus delbrueckii* subsp. *delbrueckii*	KACC 13439
*Lactobacillus delbrueckii* subsp. *delbrueckii*	KCTC 13730
*Lactobacillus acidipiscis*	KACC 12394
*Lactobacillus acidophilus*	KACC 12419
*Lactobacillus agilis*	KACC 12433
*Lactobacillus amylolyticus*	KACC 12374
*Lactobacillus amylophilus*	KACC 11430
*Lactobacillus amylovorus*	KACC 12435
*Lactobacillus brevis*	KCTC^c^ 3498
*Lactobacillus buchneri*	KACC 12416
*Lactobacillus casei*	KACC 12413
*Lactobacillus chiayiensis*	NBRC^d^ 112906
*Lactobacillus coryniformis*	KACC 12411
*Lactobacillus crustorum*	KACC 16344
*Lactobacillus curvatus*	KACC 12415
*Lactobacillus farciminis*	KACC 12423
*Lactobacillus fermentum*	KACC 11441
*Lactobacillus gallinarum*	KACC 12370
*Lactobacillus gasseri*	KCTC 3163
*Lactobacillus heilongjiangensis*	KACC 18741
*Lactobacillus helveticus*	KACC 12418
*Lactobacillus jensenii*	KCTC 5194
*Lactobacillus johnsonii*	KCTC 3801
*Lactobacillus kunkeei*	KACC 19371
*Lactobacillus lindneri*	KACC 12445
*Lactobacillus mucosae*	KACC 12381
*Lactobacillus parabuchneri*	KACC 12363
*Lactobacillus paracasei*	KCTC 3165
*Lactobacillus paraplantarum*	KACC 12373
*Lactobacillus paraplantarum*	KCTC 5045
*Lactobacillus pentosus*	KACC 12428
*Lactobacillus pentosus*	KCCM^e^ 40997
*Lactobacillus plantarum* subsp. *argentoratensis*	KACC 12404
*Lactobacillus plantarum* subsp. *plantarum*	KACC 11451
*Lactobacillus reuteri*	KCTC 3594
*Lactobacillus rhamnosus*	KCTC 3237
*Lactobacillus ruminis*	KACC 12429
*Lactobacillus sakei*	KCTC 3603
*Lactobacillus salivarius*	KCTC 3600
*Lactobacillus sanfranciscensis*	KACC 12431
*Lactobacillus zymae*	KACC 16349
*Bifidobacterium animalis* subsp. *lactis*	KACC 16638
*Bifidobacterium bifidum*	KCTC 3418
*Bifidobacterium breve*	KACC 16639
*Bifidobacterium longum* subsp. *infantis*	KCTC 3249
*Bifidobacterium longum* subsp. *longum*	KCCM 11953

^a^KACC, the Korean Agricultural Culture Collection

^b^LI, the Laboratory Isolate

^c^KCTC, the Korean Collection for Type Cultures

^d^NBRC, the NITE Biological Resource Center

^e^KCCM, the Korean Culture Center of Microorganisms

**Table 3:** Primer pairs designed in this study.

Target species	Primer name	Sequence (5'-3')	Size (bp)	Target gene	Accession no.
*L. delbrueckii* subsp. *bulgaricus*	Bulgaricus_F	TAC CGC TGT TCT GTC TCA AGG	102	YcaO-like family protein	ABJ57813.1
	Bulgaricus_R	TAT GCC TCC GTG AGC GAT CT			
*L. delbrueckii* subsp. *lactis*	Lactis_F	TTG TGC AAG AGC CAG CTG AA	113	Ser/Thr protein kinase	EGD27206.1
	Lactis_R	GCC GCC ATT ACT GAA GTG GA			
*L. delbrueckii* subsp. *delbrueckii*	Delbrueckii_F	CAT GGA ACT TCT GCG AAG GT	110	Choline kinase	KNZ37552.1
	Delbrueckii_R	TAG ATC CGG AGC TGT TCC AC			

**Table 4:** Application test of real-time PCR method to probiotic and dairy products.

Products	Type	Label claims	Detected subspecies
Products monitoring			
A1	Probiotic product (powder, Korea)	*L. delbrueckii* subsp. *bulgaricus*	*L. delbrueckii* subsp. *bulgaricus*
A2	Probiotic product (powder, Korea)	*L. delbrueckii* subsp. *bulgaricus*	*L. delbrueckii* subsp. *bulgaricus*
A3	Probiotic product (capsules, Canada)	*L. delbrueckii* subsp. *bulgaricus*	*L. delbrueckii* subsp. *bulgaricus*
A4	Probiotic product (capsules, Canada)	*L. delbrueckii* subsp. *bulgaricus*	*L. delbrueckii* subsp. *bulgaricus*
A5	Probiotic product (powder, Korea)	*L. delbrueckii* subsp. *bulgaricus*	*L. delbrueckii* subsp. *bulgaricus*
A6	Probiotic product (capsules, Korea)	*L. bulgaricus*	*L. delbrueckii* subsp. *bulgaricus*
A7	Probiotic product (capsules, USA)	*L. bulgaricus*	*L. delbrueckii* subsp. *bulgaricus*
A8	Probiotic product (capsules, USA)	*L. bulgaricus*	*L. delbrueckii* subsp. *bulgaricus*
A9	Probiotic product (powder, Korea)	*L. bulgaricus*	*L. delbrueckii* subsp. *bulgaricus*
A10	Probiotic product (powder, Korea)	*L. bulgaricus*	*L. delbrueckii* subsp. *bulgaricus*
A11	Probiotic product (capsules, Canada)	*L. bulgaricus*	*L. delbrueckii* subsp. *bulgaricus*
A12	Probiotic product (powder, Korea)	*L. bulgaricus*	*L. delbrueckii* subsp. *bulgaricus*
A13	Probiotic product (capsules, Canada)	*L. bulgaricus*	*L. delbrueckii* subsp. *bulgaricus*
A14	Probiotic product (powder, Korea)	*L. bulgaricus*	*L. delbrueckii* subsp. *bulgaricus*
A15	Probiotic product (powder, Korea)	*L. bulgaricus*	*L. delbrueckii* subsp. *bulgaricus*
B1	Dairy product (yogurt, Korea)	*L. bulgaricus*	*L. delbrueckii* subsp. *bulgaricus*
B2	Dairy product (yogurt, Korea)	*L. bulgaricus*	*L. delbrueckii* subsp. *bulgaricus*
B3	Dairy product (yogurt, Korea)	*L. bulgaricus*	*L. delbrueckii* subsp. *bulgaricus*
B4	Dairy product (yogurt, Korea)	*L. bulgaricus*	*L. delbrueckii* subsp. *bulgaricus*
B5	Dairy product (yogurt, Korea)	*L. bulgaricus*	*L. delbrueckii* subsp. *bulgaricus*
B6	Dairy product (yogurt, Korea)	*L. bulgaricus*	*L. delbrueckii* subsp. *bulgaricus*
B7	Dairy product (yogurt, Korea)	Lactic acid bacteria	*L. delbrueckii* subsp. *bulgaricus*
B8	Dairy product (yogurt, Korea)	Lactic acid bacteria	*L. delbrueckii* subsp. *bulgaricus*
B9	Dairy product (yogurt, Korea)	Lactic acid bacteria	*L. delbrueckii* subsp. *bulgaricus*
B10	Dairy product (yogurt, Korea)	Lactic acid bacteria	*L. delbrueckii* subsp. *bulgaricus*
B11	Dairy product (yogurt, Korea)	Lactic acid bacteria	*L. delbrueckii* subsp. *bulgaricus*
Identification of isolates			
I1~I4	Raw milk (cow's milk, Korea)	Unknown isolates	*L. delbrueckii* subsp. *lactis*
I5~I8	Raw milk (cow's milk, Korea)	Unknown isolates	*L. delbrueckii* subsp. *lactis*
I9~I17	Raw milk (cow's milk, Korea)	Unknown isolates	*L. delbrueckii* subsp. *lactis*
I18~I21	Dairy product (powder, Korea)	Unknown isolates	*L. delbrueckii* subsp. *bulgaricus*
I22~I57	Dairy product (yogurt, Korea)	Unknown isolates	*L. delbrueckii* subsp. *bulgaricus*
I58~I64	Dairy product (yogurt, Korea)	Unknown isolates	*L. delbrueckii* subsp. *bulgaricus*
